# T Cell Immunosenescence in Inflammatory Skin Diseases: Pathogenesis and Therapeutic Targets

**DOI:** 10.1111/acel.70527

**Published:** 2026-04-28

**Authors:** Conghui Liu, Ming Yang, Fugang Xiao, Jinrong Zeng

**Affiliations:** ^1^ Department of Dermatology The Third Xiangya Hospital, Central South University Changsha Hunan Province China; ^2^ Department of Dermatology The Second Xiangya Hospital, Hunan Key Laboratory of Medical Epigenomics, Central South University Changsha Hunan Province China

**Keywords:** inflammatory skin diseases, SASP, signal transduction, T cell immunosenescence, targeted therapy

## Abstract

T cell immunosenescence refers to the progressive functional decline of T lymphocytes with aging, characterized by the phenotypic markers, mitochondrial dysfunction, and the senescence‐associated secretory phenotype (SASP), representing a pivotal aspect of overall immune aging. This review systematically elucidates the critical role of T cell immunosenescence in the pathogenesis of common inflammatory skin diseases, including psoriasis, atopic dermatitis, rosacea, and seborrheic dermatitis. Senescent T cells drive the production of a disease‐specific SASP via internally dysregulated signaling networks such as NF‐κB, JAK‐STAT, p38 MAPK, and PI3K‐Akt‐mTOR pathways, thereby shaping and sustaining a chronic cutaneous inflammatory microenvironment that promotes disease chronicity and recurrence. Furthermore, this review summarizes current therapeutic strategies targeting these senescence‐associated pathways and SASP components, discussing both biological agents and small molecule inhibitors. Finally, we propose future research directions focusing on the direct targeting of senescent T cells or their upstream regulatory hubs to achieve deep disease remission and overcome therapeutic resistance.

## Graphical abstract

Immunosenescent T cells promote inflammatory skin diseases such as psoriasis, atopic dermatitis, rosacea, and seborrheic dermatitis via hyperactive signaling networks and SASP secretion. Consequently, intercepting downstream SASP, inhibiting internal pathways, or utilizing senolytics represents promising therapeutic interventions.

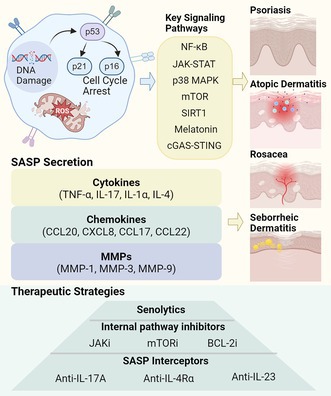

## 1 Introduction

T cell immunosenescence refers to a series of age‐associated progressive and degenerative changes in T lymphocytes at cellular, molecular, and functional levels, leading to a gradual decline in immune competence (Mittelbrunn and Kroemer 2021; Liu et al. 2023). This process constitutes a pivotal component of overall immunosenescence, with senescent T cells serving as biological markers of immune system deterioration (Hu et al. 2026). Immunosenescent T cells exhibit distinct phenotypic signatures, characterized by the downregulation of costimulatory molecules CD27 and CD28, concomitant with the upregulation of CD57 and KLRG‐1 (Lian et al. 2020). Furthermore, mitochondrial dysfunction, the senescence‐associated secretory phenotype (SASP), and telomere attrition are prominent hallmarks of these senescent cells (Mittelbrunn and Kroemer 2021).

Inflammatory skin diseases represent a class of chronic, non‐communicable disorders driven by immune dysregulation, characterized primarily by persistent inflammation and aberrant immune responses that result in cutaneous tissue damage (Ujiie et al. 2022). Importantly, beyond cutaneous manifestations, these conditions are associated with multiple comorbidities, significantly impairing patients' quality of life and imposing a substantial global disease burden (Griffiths et al. 2021; Williamson et al. 2020; Ferreira et al. 2020). Notably, immunosenescent T cells exert a pivotal pathogenic role in inflammatory skin diseases such as psoriasis, atopic dermatitis (AD), rosacea, and seborrheic dermatitis (SD) by interacting with resident keratinocytes via the SASP and reactive oxygen species (ROS), thereby facilitating the establishment and maintenance of the cutaneous inflammatory microenvironment.

However, the contribution of immunosenescent T cells to the pathogenesis of inflammatory dermatoses remains incompletely elucidated. To systematically address this gap, we conducted a comprehensive literature search across PubMed, Web of Science, and Scopus for peer‐reviewed English articles published up to February 2026. The search strategy utilized specific combinations of keywords and MeSH terms, including (“T cell immunosenescence” OR “senescent T cells” OR “immune aging”) AND (“psoriasis” OR “atopic dermatitis” OR “rosacea” OR “seborrheic dermatitis”) alongside mechanistic terms such as (“signaling pathways” OR “SASP” OR “mitochondrial dysfunction”) and (“targeted therapy” OR “senolytics”). Based on this rigorous literature selection, this review summarizes the core hallmarks of T cell immunosenescence and explicitly elucidates the pathogenic role of associated signaling networks in common inflammatory dermatoses. Furthermore, it explores the therapeutic potential of senescence‐associated targets, providing a conceptual framework for future mechanistic investigations and the development of targeted therapeutic strategies.

## 2 Molecular Drivers and Phenotypic Markers of Senescent T Cells

T cell immunosenescence is a multifaceted phenomenon encompassing lymphoid organ remodeling and complex regulatory mechanisms at the cellular level (Accardi and Caruso 2018). Although the full spectrum of immunosenescent alterations remains incompletely characterized, multiple studies have consistently documented distinct phenotypic changes, including variations in nuclear and membrane protein markers, the SASP, mitochondrial dysfunction, TCR repertoire contraction, naive‐memory imbalance, and epigenetic modifications of the TSC1/TSC2 complex.

### 2.1 The Phenotypic Markers of Senescent T Cells

The expression of markers associated with T cell immunosenescence follows a tightly regulated cascade progressing from molecular damage to functional decline (Table 1). Endogenous stress signals such as telomere attrition, DNA damage, or oxidative stress typically initiate this process by activating the transcription factor p53 via the ATM/ATR pathway. Subsequently, p53 upregulates the cyclin‐dependent kinase inhibitor p21^CIP1^ to hinder Rb protein phosphorylation through the inhibition of the Cyclin E/CDK2 complex, thereby arresting cells in the G1 phase. Concurrently, senescence‐associated epigenetic alterations induce sustained expression of p16^INK4a^, reinforcing Rb‐E2F pathway‐mediated cell cycle arrest via the inhibition of Cyclin D/CDK4/6 complexes; collectively, these mechanisms drive T cells into a state of irreversible proliferative senescence (Sun and Chen 2025).

**TABLE 1:** Phenotypic markers defining T cell senescence and related states.

Category	Markers	Functional role	References
**Senescence**			
Upstream activator	p53	Master regulator activated by persistent DNA damage; Initiates the senescence program.	Hu et al. (2026); Sun and Chen (2025)
Core arrest mediators	p21	Key effector of p53; Mediates G1 phase cell cycle arrest; Induces SASP	Wang, Wang, et al. (2024); Wang, Han, Elisseeff, and Demaria (2024)
	p16	Inhibits CDK4/6; Induces irreversible G1 phase cell cycle arrest; Mediates the establishment and maintenance of the senescence‐associated phenotype	Sun and Chen (2025); Wang, Han, Elisseeff, and Demaria (2024)
Surface markers (downregulated)	CD28	Results in costimulatory signaling defects and functional decline; Confers an acquired pro‐inflammatory and cytotoxic phenotype	Larbi et al. (2011)
	CD27	Absence of co‐stimulatory signals; Memory T cell dysfunction	Hendriks et al. (2000)
Surface markers (upregulated)	CD57	Definitive marker of terminal differentiation and replicative senescence; Correlating with shorter telomeres, less proliferation and higher expression of p16/p21	Hu et al. (2026); Lian et al. (2020)
	KLRG‐1	KLRG1 + Treg cells shift from immunosuppressive to pro‐inflammatory functions; Correlates with high p16 expression, restricts T cell over‐proliferation	Soto‐Heredero et al. (2025); Shi et al. (2014)
**T cell exhaustion**			
Co‐inhibitory receptors	Tim‐3	Senescence‐associated upregulation of immune checkpoints; Cooperates with PD‐1 to define the terminally exhausted T‐cell subset	Lee et al. (2016); Zhang, Ren, et al. (2024)
	TIGIT	A co‐inhibitory receptor; Its deficiency enhances the CD28‐mediated PI3K/AKT/mTOR costimulatory pathway	Panetti et al. (2025); Lai et al. (2025)
**Terminal differentiation**			
Differentiation and homing markers	CD45RA	A defining phenotype of TEMRA; Preferential homing to peripheral inflammatory sites	Carrasco et al. (2022)
	CCR7	Reduces the homing ability of T cells to lymph nodes; Regulates terminal differentiation and subset switching; Associates with telomere shortening	Sallusto et al. (1999); Fritsch et al. (2005); Paldino et al. (2025)

Abbreviations: CCR7, C‐C motif chemokine receptor 7; KLRG‐1, Killer cell lectin‐like receptor subfamily G member 1; mTOR, Mammalian target of rapamycin; PD‐1, Programmed cell death protein 1; PI3K, Phosphoinositide 3‐kinase; SASP, Senescence‐associated secretory phenotype; TEMRA, Terminally differentiated effector memory T cells re‐expressing CD45RA; TIGIT, T cell immunoreceptor with Ig and ITIM domains; Tim‐3, T‐cell immunoglobulin and mucin‐domain containing‐3.

**FIGURE 1:**
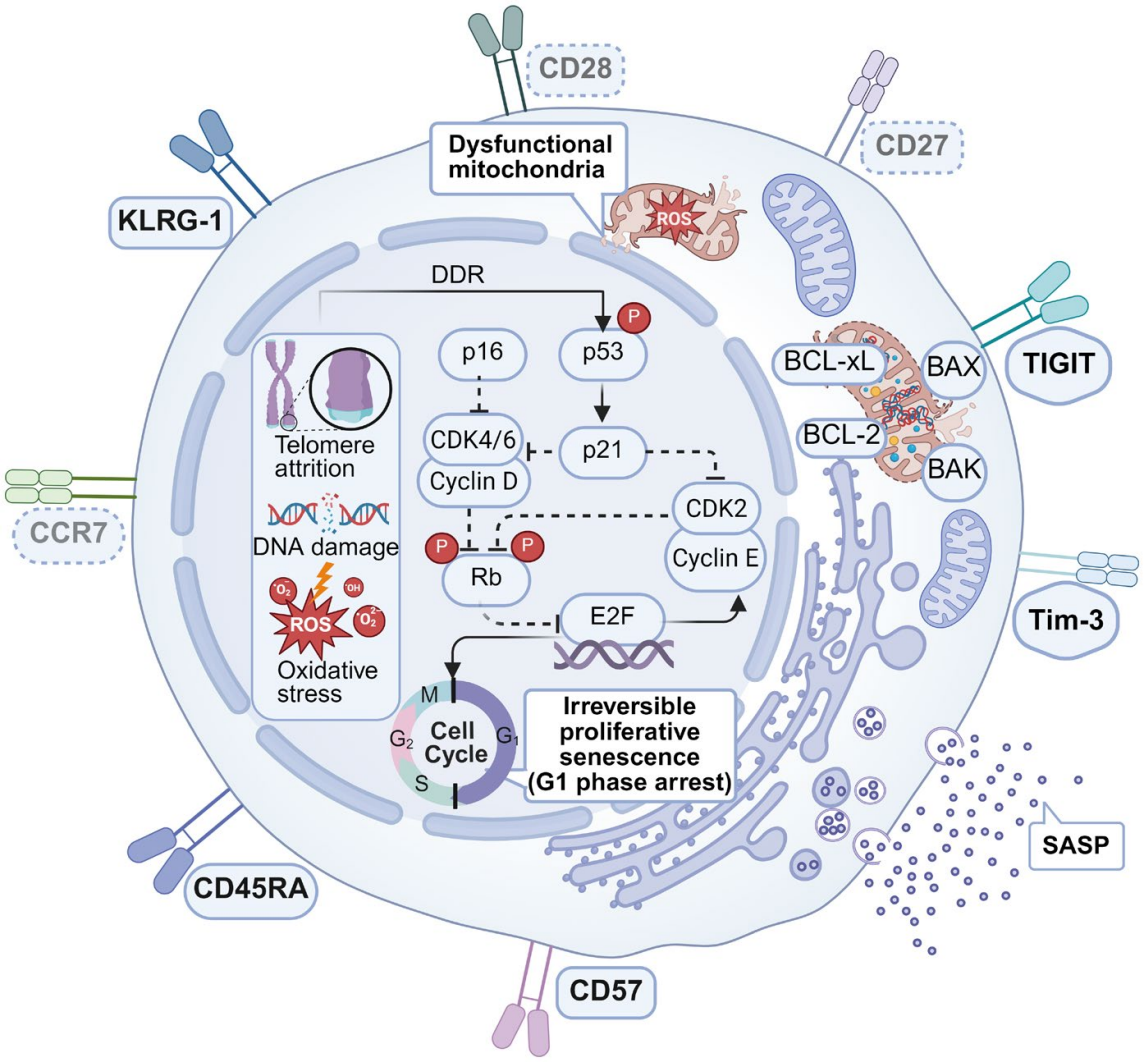
Key phenotypic and molecular hallmarks of T cell immunosenescence. The shapes of the surface markers indicate specific cellular states: Rounded rectangles represent markers of senescence, pill shapes indicate terminal differentiation, and rounded hexagons denote markers of exhaustion. Additionally, dashed borders represent the downregulation of markers (e.g., CD27, CD28), while solid borders indicate the upregulation of receptors (e.g., KLRG‐1, TIGIT). Intracellularly, telomere attrition and oxidative stress trigger the DNA damage response (DDR), activating p53/p21 and p16 pathways. These inhibit cyclin‐dependent kinases (CDKs), preventing Rb phosphorylation and sequestering E2F to enforce irreversible G1 cell cycle arrest. Mitochondrial dysfunction and SASP secretion further define the senescent state. (Created with biorender.com).

Phenotypically, immunosenescence primarily manifests as a significant loss of costimulatory molecules CD27 and CD28, alongside the upregulation of definitive senescence markers such as CD57 and KLRG‐1. While these senescent T cells frequently share phenotypic features with the CD45RA^+^CCR7^−^ terminally differentiated effector memory (TEMRA) subset, it is crucial to explicitly distinguish this senescence program from T‐cell exhaustion, which is instead characterized by the high expression of inhibitory receptors such as Tim‐3 and TIGIT (Lian et al. 2020; Paldino et al. 2025). Ultimately, unlike exhausted cells that merely exhibit compromised effector functions, senescent T cells actively drive tissue pathology through the robust release of SASP‐related factors (Wang, Wang, Han, Elisseeff, and Demaria 2024), thus impairing overall immune competence and fostering the formation of an inflammatory microenvironment (Figure 1).

### 2.2 The SASP

Upon entering irreversible proliferative senescence, T cells evolve into a persistent source of inflammatory signals, a function mediated by SASP. SASP acts as a complex secretory profile composed of proinflammatory cytokines (e.g., IL‐1α/β, IL‐4, IL‐6, TNF‐α, IFN‐α), chemokines (e.g., CCL20, CCL2, CCL5, CXCL10), growth factors (e.g., TGFβ, GDF15), matrix metalloproteinases (MMPs), and their inhibitors (TIMPs), which recruits and activates other immune cells to participate in tissue remodeling (Wang, Han, Elisseeff, and Demaria 2024).

The expression of this secretory profile is precisely orchestrated by a core signaling network: NF‐κB serves as the central transcriptional hub (Chien et al. 2011); the p38 MAPK pathway amplifies inflammatory output by enhancing SASP mRNA stability (Alspach et al. 2014); and mTOR signaling promotes the translation of membrane‐bound IL‐1α and IL‐1α‐mediated NF‐κB transcriptional activity (Laberge et al. 2015). Crucially, accumulated genomic damage such as senescence‐associated mitochondrial DNA leakage is sensed by the cGAS‐STING pathway (McArthur et al. 2018; Victorelli et al. 2023), thereby synergistically activating NF‐κB to form a positive feedback loop that initiates and sustains SASP (Dou et al. 2017).

Importantly, the senescence‐associated signaling network fundamentally diverges from homologous pathways conventionally activated in pathogenic effector cells like Th17 or Th2 subsets. Although canonical inflammatory cascades including NF‐κB, JAK‐STAT, and mTOR operate constitutively in both populations within chronic skin lesions, their driving forces contrast sharply. Exogenous stimuli like persistent antigens activate these pathways in normal pathogenic T cells to fuel clonal expansion (Waickman and Powell 2012; Song et al. 2008). Conversely, these networks in senescent T cells are perpetually driven by unresolvable endogenous stress, specifically chronic DNA damage, telomere attrition, and mitochondrial dysfunction evidenced by cGAS‐STING activation (Victorelli et al. 2023; Dou et al. 2017). This continuous endogenous damage orchestrates a robust SASP within metabolically hyperactive cells that are irreversibly arrested in the G1 phase and profoundly resistant to apoptosis (Gorgoulis et al. 2019). The specific convergence of endogenous damage‐driven signaling, permanent proliferative arrest, and apoptosis evasion ultimately establishes senescent T cells as a distinct and persistent inflammatory reservoir.

### 2.3 Mitochondrial Dysfunction

Mitochondrial dysfunction serves as a core metabolic hub driving T cell immunosenescence, amplifying cell‐autonomous defects into organism‐wide aging signals by inducing intrinsic metabolic reprogramming and triggering systemic inflammation. Dysfunctional mitochondria manifest as impaired oxidative phosphorylation (OXPHOS) and excessive ROS production, whereby impaired OXPHOS leads to the reduction of tricarboxylic acid cycle intermediates such as acetyl‐CoA, α‐ketoglutarate, and NAD+, which serve as substrates or signaling molecules for epigenetic modifications, subsequently affecting the activity of histone and DNA demethylases and potentially contributing to senescence‐associated epigenetic silencing (Zhu et al. 2022). Furthermore, treatment with ROS scavengers prevents telomere shortening in in vitro CD8 T cell subsets, explicitly demonstrating that increased mitochondrial ROS is a primary contributing factor to telomere attrition during T cell senescence (Sanderson and Simon 2017). Concurrently, the age‐related decline in mitophagy efficiency leads to the accumulation of dysfunctional mitochondria, whose leaked mtDNA and excessive ROS production continuously drive SASP expression by activating signaling pathways such as cGAS‐STING, NF‐κB, and the NLRP3 inflammasome, thereby amplifying cell‐autonomous metabolic defects into systemic inflammation (Picca et al. 2023).

### 2.4 Pre‐Senescent Alterations in T Cell Compartments

Immunosenescence constitutes a continuous and progressive biological continuum, wherein premature aging of the immune system profoundly alters the phenotype and function of non‐senescent T cell pools long before they reach terminal senescence (Goronzy and Weyand 2019). Chronic inflammatory stress combined with systemic aging induces a fundamental compositional shift characterized by a progressive depletion of the naive T cell compartment and a compensatory yet dysregulated expansion of memory subsets (Nikolich‐Žugich 2018). This structural decline diminishes the overall adaptive capacity of the cutaneous immune network. Concurrently, continuous antigenic stimulation in chronic dermatoses drives severe T cell receptor repertoire contraction among these non‐senescent effector cells. Such continuous pressure limits their immunological plasticity and promotes the accumulation of oligoclonal populations highly prone to driving targeted tissue damage (Goronzy and Weyand 2019; Xu et al. 2025). Beyond these intrinsic alterations, these functional populations are continuously subjected to the SASP generated by their fully senescent counterparts within the skin microenvironment. This persistent paracrine exposure induces metabolic rewiring and a state of pre‐senescent hyper‐reactivity (Acosta et al. 2013; Wang, Han, Elisseeff, and Demaria 2024). Consequently, the broader phenomenon of immune aging compromises the entire T cell network by predisposing these otherwise functional T cells to accelerated exhaustion and exaggerated inflammatory responses.

Beyond the aforementioned core mechanisms, T cell immunosenescence is characterized by systemic changes including thymic involution, telomere shortening, DNA methylation, and proteostasis imbalance (Mittelbrunn and Kroemer 2021). These profound phenotypic and functional changes collectively constitute the typical characteristics of senescent T cells, impacting the cutaneous immune microenvironment and thereby participating in the regulation of the pathological progression of inflammatory skin diseases.

## 3 T Cell Immunosenescence Drives Inflammatory Skin Disease Pathogenesis

### 3.1 T Cell Immunosenescence in Psoriasis

Psoriasis is a chronic inflammatory skin disease characterized by aberrant keratinocyte proliferation and Th17 cell infiltration, manifesting clinically as erythema, scaling, and plaque formation (Griffiths et al. 2021). Zhu et al. confirmed an increased proportion of characteristic senescent CD4^+^ T cell subsets expressing high levels of cell cycle inhibitory proteins p16^INK4a^ and p21^CIP1^ in the lesional skin of patients with psoriasis (Zhu et al. 2024). Šahmatova et al. highlighted that T cells in patients with psoriasis display signs of excessive immune activation and features of early immunosenescence: terminally differentiated or senescent T cells (TEMRA and CD28‐TEMRA) are present in higher proportions in patients with psoriasis and are more pronounced in patients with a longer disease duration (≥ 15 years) (Šahmatova et al. 2017). Distinct from replicative senescence, this senescence phenotype is primarily induced by the chronic inflammatory microenvironment, manifesting as a permanent arrest of cell proliferation while remaining metabolically active with potent proinflammatory secretory functions.

#### 3.1.1 IL‐23/JAK‐STAT Driven Hyperproliferation

Within the psoriatic lesional microenvironment, IL‐23 binds to the receptor complex composed of IL‐23Rα and IL‐12Rβ1 on the surface of senescent T cells, activating the coupled JAK2 and TYK2 kinases. Upon cross‐phosphorylation, they specifically phosphorylate the transcription factor STAT3. Activated STAT3 forms homodimers and translocates into the nucleus, binding to the promoter regions of IL‐17A and IL‐22 genes to drive their high transcription (Guo et al. 2023; Furtunescu et al. 2024). IL‐17A serves as the most potent inducer of the subsequent inflammatory cascade (Guo et al. 2023), while IL‐22 directly stimulates keratinocyte hyperproliferation and aberrant differentiation via the JAK1/TYK2‐STAT3 axis, resulting in hyperkeratosis and parakeratosis, which clinically manifest as typical epidermal thickening and scale formation (Furtunescu et al. 2024).

#### 3.1.2 NF‐κB/p38 MAPK Orchestrated Inflammatory Infiltration

Concurrently, the NF‐κB pathway acts as a classic central hub of inflammatory response and is continuously activated by SASP components such as TNF‐α and IL‐1β. Signal transduction leads to the activation of the IKK complex, which subsequently phosphorylates and degrades the inhibitory protein IκBα, allowing the release and rapid nuclear translocation of p50/RelA and p50/c‐Rel dimers (Sugumaran et al. 2024). These dimers not only extensively upregulate the expression of inflammatory mediators including TNF‐α, IL‐1β, IL‐6, iNOS, E‐selectin, and VCAM‐1 but also, more critically, specifically induce the production of the key chemokine CCL20 (Zhang et al. 2023; Collins et al. 1995). CCL20 binds to CCR6+ Th17 cells and dendritic cells expressing its receptor to form the CCL20‐CCR6 axis, precisely recruiting more pathogenic immune cells to the dermis to form a dense inflammatory infiltrate, causing vasodilation and tortuosity in the papillary dermis as well as massive lymphocyte accumulation, which clinically manifests as persistent erythema (Wei et al. 2024; Shi et al. 2022).

This process is deeply synergistic with the p38 MAPK pathway: p38α kinase activated by IL‐17A signaling phosphorylates the transcription factor ATF2 to promote the expression of neutrophil chemokines such as IL‐8 (CXCL8) (Rajaiya et al. 2008), which is associated with pustular psoriasis or microabscess formation; on the other hand, p38 MAPK activation directly phosphorylates the S536 site of RelA of NF‐κB independently of IKKγ or IκBα degradation, thereby enhancing its transcriptional activity (Jijon et al. 2004) and forming a positive feedback loop.

#### 3.1.3 PI3K/Akt/mTOR Mediated Metabolic Reprogramming

In immunosenescent T cells of psoriasis, hyperactivation of the PI3K/Akt/mTOR pathway serves as a core metabolic switch for acquiring and sustaining pathogenic functions. This pathway responds to SASP signals such as IL‐1β, TNF‐α, IL‐17A, and IL‐22 (Cibrian et al. 2020), where activated Akt relieves the inhibition of mTORC1 by suppressing the TSC1/TSC2 complex. Activated mTORC1 subsequently phosphorylates downstream effectors 4E‐BP1 and S6K1, significantly enhancing protein translation efficiency to robustly synthesize characteristic SASP components like IL‐17A and IFN‐γ that drive aberrant keratinocyte proliferation and inflammation (Mercurio et al. 2021). Concurrently, sustained Akt activation leads to reduced antioxidant capacity by inhibiting FOXO3a transcriptional activity, resulting in ROS accumulation which stabilizes and activates p53 (Wrone‐Smith et al. 1997), while mTORC1 also directly promotes p53 translation and interferes with its ubiquitination and degradation (Astle et al. 2012; Nogueira et al. 2008), collectively leading to the sustained high expression of the cell cycle inhibitor p21^WAF1/Cip1^ (Mercurio et al. 2021; Miyauchi et al. 2004). This Akt/mTOR/p53/p21 axis directly dictates the entry of T cells into a growth‐arrested yet hypersecretory senescent state at the molecular level.

Furthermore, mTORC2 fully activates Akt by phosphorylating its Ser473 site, which not only positively amplifies the aforementioned pathway to form a metabolic positive feedback loop but also reinforces the long‐term survival and adaptability of senescent T cells by phosphorylating and inhibiting pro‐apoptotic proteins such as BAD (Mercurio et al. 2021) and inducing FOXO protein phosphorylation to prevent autophagy (Ballesteros‐Álvarez and Andersen 2021), thereby exacerbating the chronic progression of the disease.

#### 3.1.4 Oxidative Stress Driving SIRT1/Melatonin Dysfunction

In psoriatic senescent T cells, excessive ROS not only acts as a second messenger to directly activate stress pathways such as p38 MAPK (Liu et al. 2022) but, more critically, impairs the NAD + ‐dependent Sirtuin pathway, leading to a significant decline in the activity of the deacetylase SIRT1 (Zhao et al. 2021). SIRT1 functional exhaustion attenuates its deacetylation‐mediated inhibition of two core transcription factors, the NF‐κB p65 subunit and STAT3 (Sun et al. 2021; Xu et al. 2019). Acetylation of p65 enhances its transcriptional activity, directly driving the expression of factors such as TNF‐α, IL‐6, and CCL20 (Sugumaran et al. 2024; Zhang et al. 2023; Collins et al. 1995); while STAT3 acetylation synergizes with IL‐23R signaling to maximize IL‐17A transcription (Furtunescu et al. 2024). This mechanism directly relieves the negative regulation on the psoriatic IL‐23/Th17 inflammatory axis and the NF‐κB cascade at the epigenetic level, forming a positive feedback loop that exacerbates inflammatory transcription and drives epidermal hyperplasia and chronic inflammatory infiltration (Guo et al. 2023).

Similarly, the antioxidant protective role of the endogenous melatonin pathway is impaired in senescent T cells. Melatonin originally scavenges ROS and inhibits NLRP3 inflammasome activation via its MT1/MT2 receptors (Zheng et al. 2023). The attenuation of this pathway significantly relieves the inhibition of the NLRP3 inflammasome, leading to increased caspase‐1‐mediated maturation and release of IL‐1β and IL‐18 (Gupta et al. 2025). IL‐1β is a potent neutrophil chemoattractant and pyrogen that is highly correlated with pathological features such as neutrophil microabscess formation, acute inflammatory flares, and systemic symptoms in psoriasis, especially the pustular type (Johnston et al. 2017).

#### 3.1.5 Cytosolic DNA Sensing via cGAS‐STING

Furthermore, genomic instability and mtDNA leakage caused by telomere dysfunction and oxidative damage in senescent T cells can be recognized by the cytosolic DNA sensor cGAS‐STING pathway. cGAS catalyzes the generation of 2′3′‐cGAMP, activating the endoplasmic reticulum‐anchored protein STING, which subsequently recruits and phosphorylates the kinase TBK1, ultimately leading to IRF3 phosphorylation, dimerization, and nuclear translocation. IRF3 synergizes with NF‐κB to drive the massive production of Type I Interferons (Sun et al. 2024). IFN‐α binds to the type I interferon receptor (IFNAR) on the surface of senescent T cells in an autocrine/paracrine manner, activating the JAK1/TYK2‐STAT1/STAT2/STAT3 pathway (Furtunescu et al. 2024), thereby integrating endogenous damage signals of senescent T cells into the SASP network and endowing psoriasis with autoinflammatory characteristics.

**FIGURE 2:**
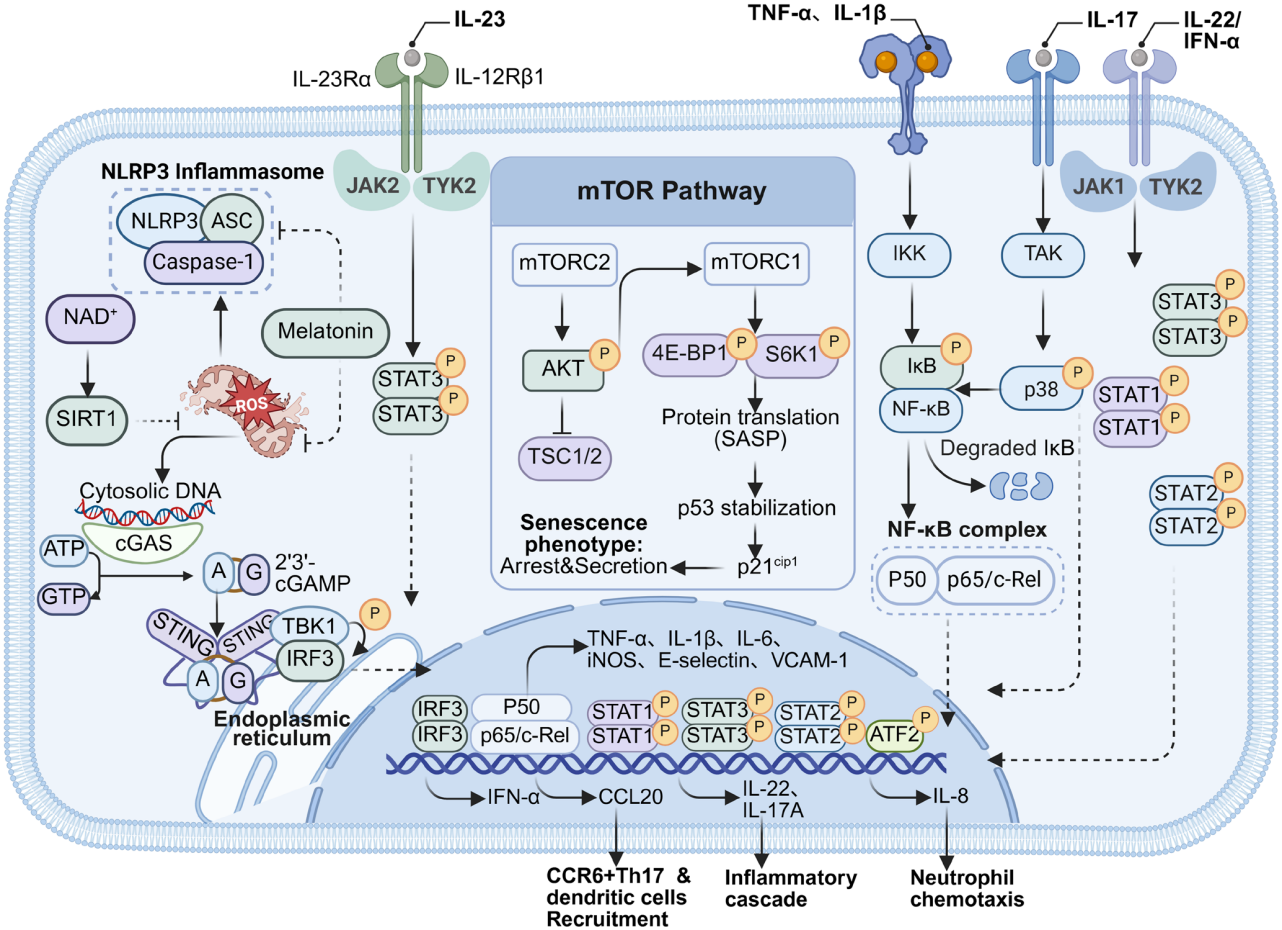
Signaling networks of immunosenescent T cells in psoriasis. Extracellular inflammatory cytokines and intracellular metabolic stress coordinate to drive the senescence‐associated secretory phenotype (SASP) in psoriatic T cells. Ligand binding of interleukin‐23 (IL‐23), IL‐17, and tumor necrosis factor‐α (TNF‐α) activates Janus kinase (JAK)–signal transducer and activator of transcription (STAT) and nuclear factor‐κB (NF‐κB) cascades, while the mammalian target of rapamycin (mTOR) pathway stabilizes p53 to enforce cell cycle arrest. Concurrently, cytosolic DNA and mitochondrial reactive oxygen species (ROS) trigger the cGAS‐STING sensor and NLRP3 inflammasome, respectively. These convergent signaling axes induce the phosphorylation and nuclear translocation of key transcription factors (STATs, NF‐κB, and IRF3), orchestrating the expression of inflammatory mediators (CCL20, IL‐8) that sustain neutrophil chemotaxis and CCR6+ cell recruitment. (Created with biorender.com).

In summary, the pathogenic role of senescent T cells in psoriasis stems from an integrated signaling network that culminates in the production and secretion of disease‐specific SASP. The JAK‐STAT and NF‐κB pathways are central to programming SASP transcription (Tomar et al. 2023; Souto‐Silva et al. 2025); p38 MAPK acts to synergistically amplify the signal (Wang, Han, Huang, et al. 2024); the PI3K/Akt/mTOR pathway provides metabolic and translational support (Souto‐Silva et al. 2025); while accompanying mitochondrial dysfunction and oxidative stress compromise endogenous protective pathways such as SIRT1 and melatonin, weakening the epigenetic and metabolic homeostatic regulation of key proinflammatory transcription factors (Słuczanowska‐Głabowska et al. 2023; Shen et al. 2024). Concurrently, the cGAS‐STING pathway directly converts senescence‐associated endogenous danger signals into a type I interferon response (Willemsen et al. 2021). This network drives the sustained production of SASP centered on IL‐17A and CCL20, directly leading to aberrant keratinocyte proliferation, dermal inflammatory infiltration, and vascular changes, thereby establishing the core driving position of senescent T cells in the pathology of psoriasis (Figure 2).

### 3.2 T Cell Immunosenescence in AD

AD is a chronic inflammatory skin disease driven by a core Th2‐type immune response, manifesting primarily as skin barrier dysfunction and intense pruritus (Weidinger et al. 2018). Studies indicate that the AD pruritus‐associated cytokine IL‐31 induces p21^Cip1^ expression, thereby inducing a senescence phenotype (Kasraie et al. 2013). Photoaging caused by long‐term ultraviolet radiation acts as a factor that can aggravate barrier dysfunction and oxidative stress in AD, and can also induce cellular p53, p21, and p16 senescence phenotypes (Ansary et al. 2021; Beattie et al. 2005; Park et al. 2023).

#### 3.2.1 Th2/JAK‐STAT‐Driven Barrier Disruption and Pruritus

Within the chronic inflammatory environment of AD, immunosenescent T cells significantly contribute to disease chronicity by establishing pathogenic cross‐talk with the epidermis via their aberrantly activated signaling networks. Specifically, IL‐4 binds to the IL‐4Rα/γc receptor on the cell surface, activating JAK1 and JAK3, which subsequently specifically phosphorylate STAT6. Activated STAT6 dimers translocate into the nucleus and directly initiate high transcription of IL‐4 and IL‐13 genes (Weidinger et al. 2018; Huang et al. 2022), establishing the foundation for the Th2‐type SASP profile. Through paracrine cross‐talk, these highly secreted IL‐4 and IL‐13 cytokines subsequently act on the type II receptor IL‐4Rα/IL‐13Rα1 on the surface of adjacent keratinocytes. Within the keratinocytes, through the JAK‐STAT6 pathway, they inhibit positive transcription factors (e.g., Ovol‐1, Grhl3) for key barrier proteins such as filaggrin, loricrin, and involucrin within the keratinocyte nucleus (D'Avino et al. 2026; Furue 2020) and concurrently upregulate the expression of specific proteases (e.g., serine proteases, kallikreins) and deubiquitinases (Yasuda et al. 2016), accelerating the degradation of synthesized barrier protein precursors. This dual mechanism of inhibiting synthesis and accelerating degradation driven by T cell‐derived SASP promotes the disruption of the physical skin barrier (Hülpüsch et al. 2024), clinically manifesting as significant xerosis, scaling, and a dramatic increase in transepidermal water loss.

Damaged keratinocytes subsequently release the alarmin thymic stromal lymphopoietin (TSLP), which conversely activates the JAK1/JAK2‐STAT5 axis within senescent T cells via its receptor, further reinforcing the transcription and secretion of IL‐4/IL‐13 (Huang et al. 2022), forming a self‐sustaining inflammatory cycle of immune attack, barrier disruption, and alarm amplification. Notably, IL‐31, a key factor mediating pruritus, also promotes its own production via the JAK1/2‐STAT1/3/5 pathway, forming an autocrine loop (Guttman‐Yassky et al. 2023), which explains the direct association between senescent T cells and the chronic, refractory pruritus symptoms of AD.

#### 3.2.2 p38 MAPK/NF‐κB Fueling Th2 Loops and Infiltration

The Th2 polarization program and its barrier‐disrupting effects are further amplified via pathways including p38 MAPK and NF‐κB. The p38α kinase, activated by signals such as oxidative stress, not only directly enhances inflammatory gene transcription but also directly stimulates the transcriptional activation of STAT6 to regulate IL‐4‐induced gene expression (Pesu et al. 2002), thereby reinforcing the Th2 transcriptional program and establishing a positive feedback loop linking cellular stress to specific inflammatory output. Concurrently, the NF‐κB pathway is continuously activated by TNF‐α and TSLP (Seo et al. 2024), driving the production of general inflammatory factors such as TNF‐α as well as specific chemokines including CCL17 and CCL22 (Wang, Liu, et al. 2025; Jeong et al. 2010). These chemokines are responsible for recruiting CCR4+ Th2 cells and eosinophils to the skin, resulting in the characteristic eosinophilic infiltration observed in AD lesions (Matsuo et al. 2019).

#### 3.2.3 mTORC1/mTORC2 Dismantling Barrier

The aberrantly activated mTOR pathway in senescent T cells drives AD skin barrier disruption and inflammation chronicity via a dual mechanism. On one hand, hyperactivation of the mTORC1 complex not only directly inhibits the expression and processing of filaggrin via the mTORC1/AKT1/cathepsin H (CTSH) axis (Naeem et al. 2017) but also functions as a key negative regulatory switch for autophagy. This inhibition of autophagic flux leads to the accumulation of the autophagy receptor p62, which subsequently aberrantly activates pathways such as Nrf2 and NF‐κB, exacerbating oxidative stress and inflammation (Sukseree et al. 2021; Hou et al. 2020). These processes collectively severely compromise keratinocyte homeostasis and differentiation, dismantling the physical skin barrier. On the other hand, the mTORC2 complex consolidates Th2 polarization and promotes senescent T cell survival by phosphorylating Akt at the Ser473 site (Roy et al. 2023), thereby maintaining their sustained pathogenic output. Thus, mTORC1 and mTORC2 act synergistically to disrupt epithelial barrier integrity and maintain the pool of inflammatory cells, collectively driving the chronicity of AD.

#### 3.2.4 SIRT1/Melatonin Deficiency Compromising Barrier and Immunity

Oxidative stress‐induced decline in SIRT1 activity within immunosenescent T cells severely compromises skin barrier function. In keratinocytes, SIRT1 directly upregulates filaggrin expression by activating the aryl hydrocarbon receptor pathway. Studies confirm that epidermal SIRT1 deficiency induces AD‐like lesions and exacerbates allergen sensitivity (Ming et al. 2015). Notably, reduced SIRT1 expression is also observed in filaggrin‐deficient models, while antioxidant intervention simultaneously restores levels of SIRT1 and barrier junction proteins such as E‐cadherin and occludin (Nakai et al. 2012), indicating that the oxidative stress‐SIRT1 depletion‐barrier disruption axis constitutes a vicious cycle driving AD. Impairment of the melatonin pathway in immunosenescent T cells directly attenuates its intrinsic antioxidant and anti‐inflammatory functions, resulting in a failure to effectively inhibit the excessive production of Th2‐type cytokines and IgE (Marseglia et al. 2014). Clinical trials demonstrate that melatonin supplementation alleviates sleep disorders and reduces disease severity in patients with AD (Chang et al. 2016).

**FIGURE 3:**
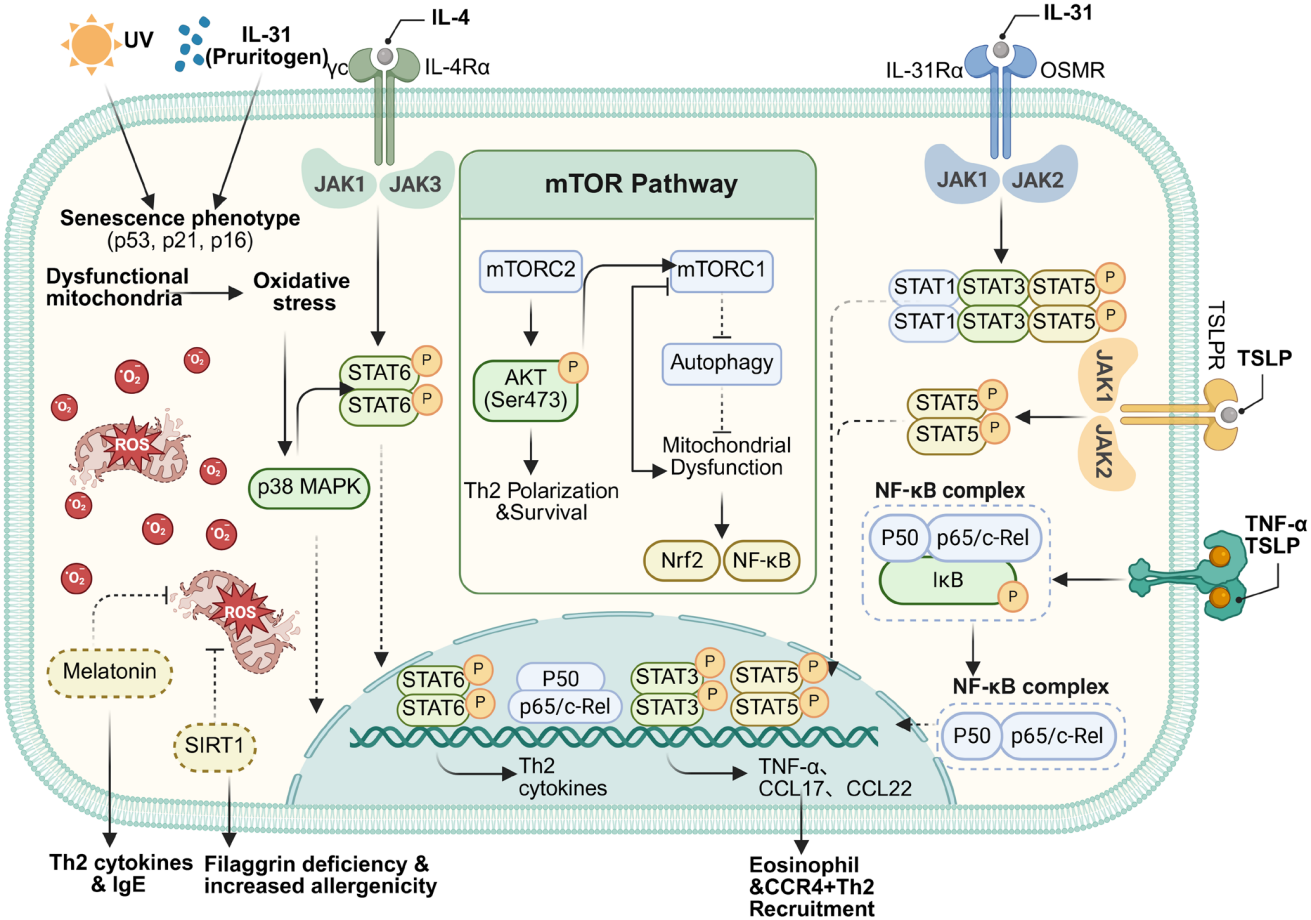
Signaling networks of immunosenescent T cells in AD. Extracellular pruritogens and inflammatory cytokines synergize with intracellular metabolic stress to drive the pathogenic Th2 phenotype in atopic dermatitis (AD). Engagement of IL‐4, IL‐31, and thymic stromal lymphopoietin (TSLP) receptors activates Janus kinase (JAK) signaling cascades, resulting in the phosphorylation of signal transducer and activator of transcription (STAT) factors (STAT6, STAT3, and STAT5). Concurrently, the mammalian target of rapamycin (mTOR) pathway promotes Th2 polarization via AKT (protein kinase B) and inhibits autophagy, exacerbating mitochondrial dysfunction. The resultant accumulation of reactive oxygen species (ROS), amplified by ultraviolet (UV) exposure, activates p38 mitogen‐activated protein kinase (MAPK) and nuclear factor‐κB (NF‐κB), overriding sirtuin 1 (SIRT1)–mediated antioxidant defenses. The nuclear convergence of these transcription factors orchestrates the expression of Th2 cytokines, tumor necrosis factor‐α (TNF‐α), and chemokines (CCL17, CCL22), which collectively induce filaggrin deficiency, immunoglobulin E (IgE) synthesis, and the recruitment of eosinophils and CCR4+ Th2 cells. (Created with biorender.com).

Within the immunosenescent T cell network of AD, the role of the cGAS‐STING pathway is relatively limited compared to that in psoriasis. The JAK‐STAT pathway initiates the cascade, driving Th2 polarization and the secretion of characteristic SASP components such as IL‐4/IL‐13 (Li et al. 2021); subsequently, the p38 MAPK and NF‐κB pathways synergistically amplify inflammatory signals and mediate eosinophil chemotaxis (Park et al. 2015; Liu et al. 2011). Concurrently, the mTOR pathway directly dismantles the physical skin structure by inhibiting autophagy and impairing barrier protein processing (Hou et al. 2020; Cibrian et al. 2020). Furthermore, the functional impairment of SIRT1 and melatonin pathways induced by oxidative stress further relieves key endogenous inhibition of inflammation and oxidative damage (Ming et al. 2015; Jaworek et al. 2021). These pathways collectively translate the aberrant activation state of senescent T cells into the chronic pathological progression of AD, characterized by persistent Th2 inflammation, barrier dysfunction, and intense pruritus (Figure 3).

### 3.3 T Cell Immunosenescence in Rosacea

Rosacea is characterized by recurrent flushing, persistent erythema, inflammatory papules/pustules, and telangiectasia (van Zuuren 2017), with pathogenesis primarily involving neurovascular dysregulation and aberrant innate immune activation (van Zuuren et al. 2021). While the exact mechanisms linking aging and rosacea remain poorly understood, recent studies indicate that the SIRT7‐TLR2‐NF‐κB axis plays a key role in the pathogenesis of rosacea in the elderly population (Li et al. 2022).

In the pathogenesis of rosacea, the NF‐κB pathway within immunosenescent T cells serves as a crucial signaling hub for the inflammatory response, strongly triggered by ultraviolet radiation, neuropeptides such as Substance P and CGRP, and the TLR2/MyD88 signaling axis activated following skin barrier compromise (Yang et al. 2024). Activated NF‐κB drives these senescent T cells to secrete a potent SASP (IL‐1β, IL‐6, TNF‐α, CXCL8), subsequently triggering epidermal keratinocytes to produce the antimicrobial peptide LL37 (Shen et al. 2023; Qi et al. 2024). Importantly, LL37 not only forms proinflammatory complexes that directly cause vascular damage but also further activates the MAPK pathway, leading to the phosphorylation of ERK1/2 and p38 kinases, thereby establishing a positive feedback loop with NF‐κB to continuously amplify inflammatory signals (Yang et al. 2024). The inflammatory cytokine network regulated by this synergistic immune‐epithelial mechanism ultimately leads to characteristic vascular reactions and tissue damage, manifesting as the formation of persistent erythema, papules, and pustules.

Crosstalk exists between the JAK‐STAT signaling pathway of immunosenescent T cells and TLR2 signaling. TLR2 activation further activates the JAK‐STAT pathway, thereby regulating Th1 and Th17 cell differentiation and stimulating the production of pro‐angiogenic factors such as VEGF (Liu et al. 2019; Hu et al. 2025), linking immune dysregulation with vascular pathology. Concurrently, this pathway directly transduces signals from the core pruritic factor IL‐31, mediating pruritus (Erickson et al. 2021).

In immunosenescent T cells, mTORC1 forms a positive feedback loop with LL37, where LL37 activates mTORC1 by binding to TLR2, and activated mTORC1 conversely promotes LL37 generation, leading to massive LL37 accumulation that continuously stimulates inflammatory responses, constituting a vicious cycle (Deng et al. 2021).

**FIGURE 4:**
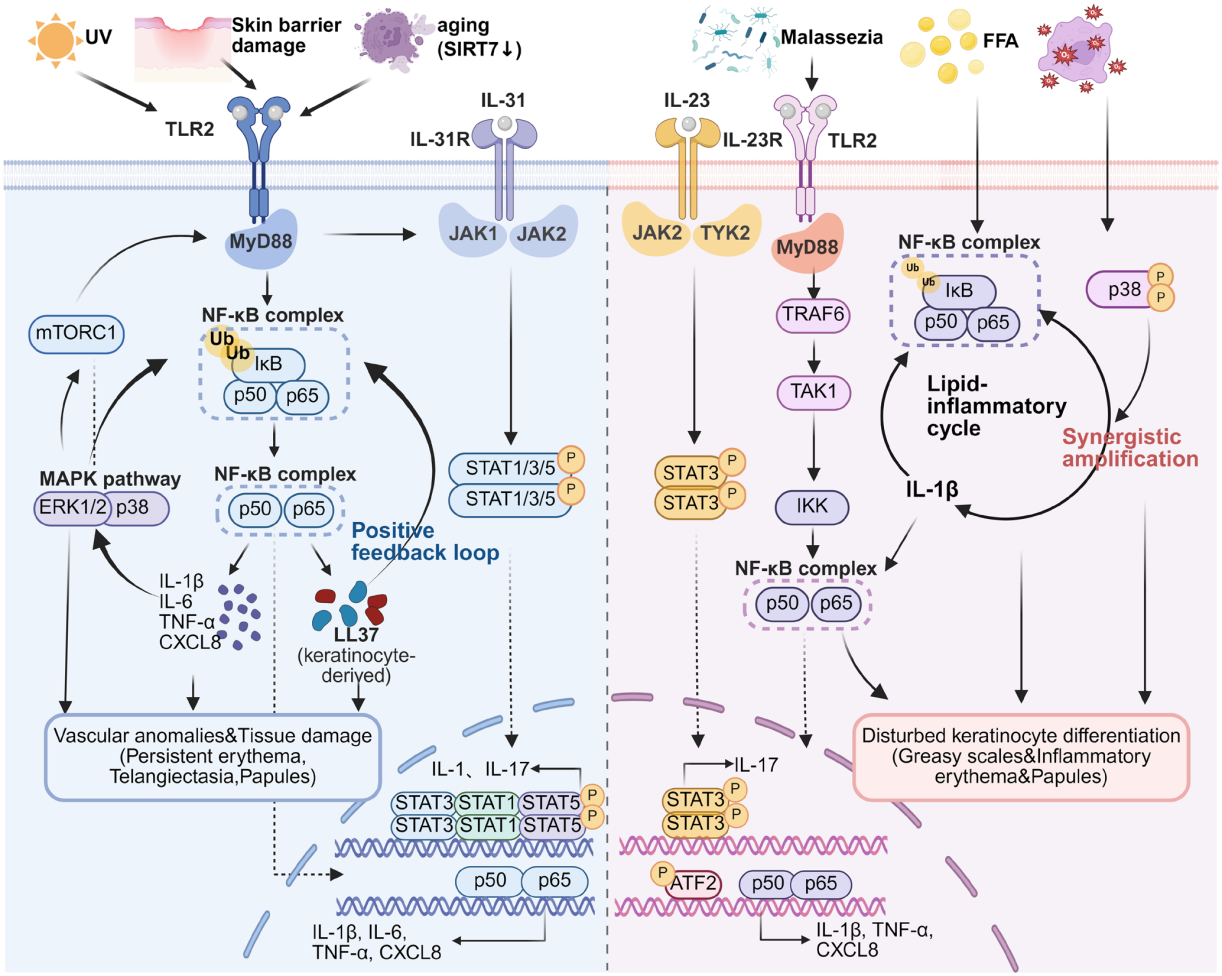
Signaling networks of immunosenescent T cells in Rosacea and SD. Distinct environmental and intrinsic stressors drive pathogenic signaling in Rosacea versus Seborrheic Dermatitis (SD). In Rosacea (left panel), ultraviolet (UV) radiation, skin barrier damage, and aging‐associated sirtuin 7 (SIRT7) downregulation activate Toll‐like receptor 2 (TLR2) and interleukin‐31 (IL‐31) axes. The TLR2–myeloid differentiation primary response 88 (MyD88) cascade triggers the nuclear factor‐κB (NF‐κB) complex and mitogen‐activated protein kinase (MAPK) pathways (ERK1/2, p38), establishing a positive feedback loop with cathelicidin (LL37) that perpetuates vascular anomalies and persistent erythema. In SD (right panel), *Malassezia* colonization and free fatty acids (FFA) engage TLR2 and IL‐23 receptors, initiating a lipid‐inflammatory cycle involving IL‐1β. Convergent signaling via Janus kinase (JAK)–signal transducer and activator of transcription 3 (STAT3) and oxidative stress–mediated p38 amplification promotes the nuclear translocation of NF‐κB and activating transcription factor 2 (ATF2). This transcriptional program drives disturbed keratinocyte differentiation, resulting in the clinical presentation of greasy scales and inflammatory papules. (Created with biorender.com).

In summary, the core inflammatory response initiated by NF‐κB and p38 MAPK in immunosenescent T cells, together with the network constituted by the mTORC1‐LL37 positive feedback loop and the JAK‐STAT pathway in immunosenescent T cells, collectively contribute to the chronic inflammation and vascular abnormalities in rosacea (Figure 4).

### 3.4 T Cell Immunosenescence in SD

SD is a chronic inflammatory skin disease characterized by a core Th17/Th1‐type inflammatory response aberrantly triggered by skin‐resident *Malassezia* species (Ungar et al. 2025).

In SD, immunosenescent T cells exacerbate disease progression via an aberrant interplay with the epidermal compartment. Initially, skin‐resident keratinocytes sense *Malassezia* and actively secrete IL‐23. Concurrently, immunosenescent T cells intrinsically sense fungal metabolites. Within these senescent T cells, the upstream TLR2/MyD88 signaling axis triggers IKK complex activation, subsequently leading to the degradation of the inhibitory protein IκBα and releasing p65/p50 dimers for nuclear translocation, with the NF‐κB pathway acting as a core switch. Driven by this internal signaling and the keratinocyte‐derived IL‐23, senescent T cells robustly produce IL‐17A via the JAK2/TYK2‐STAT3 axis, alongside a potent SASP that includes IL‐1β, TNF‐α, and CXCL8 (Jia et al. 2024; Wang, Han, Elisseeff, and Demaria 2024). This T cell‐derived inflammatory profile subsequently acts back on the epidermis, with IL‐17A disrupting keratinocyte differentiation to cause characteristic greasy scales (Jia et al. 2024), and the SASP promoting clinical inflammatory erythema and papules. Moreover, the p38 MAPK pathway responds to oxidative stress and synergizes with NF‐κB to amplify inflammatory signals, reinforcing the persistence of inflammation (Briganti and Picardo 2003; Faergemann et al. 2001). Furthermore, the increase in free fatty acids in SD also activates NF‐κB and promotes IL‐1β production, generating a lipid‐inflammation vicious cycle (Adalsteinsson et al. 2020).

**FIGURE 5:**
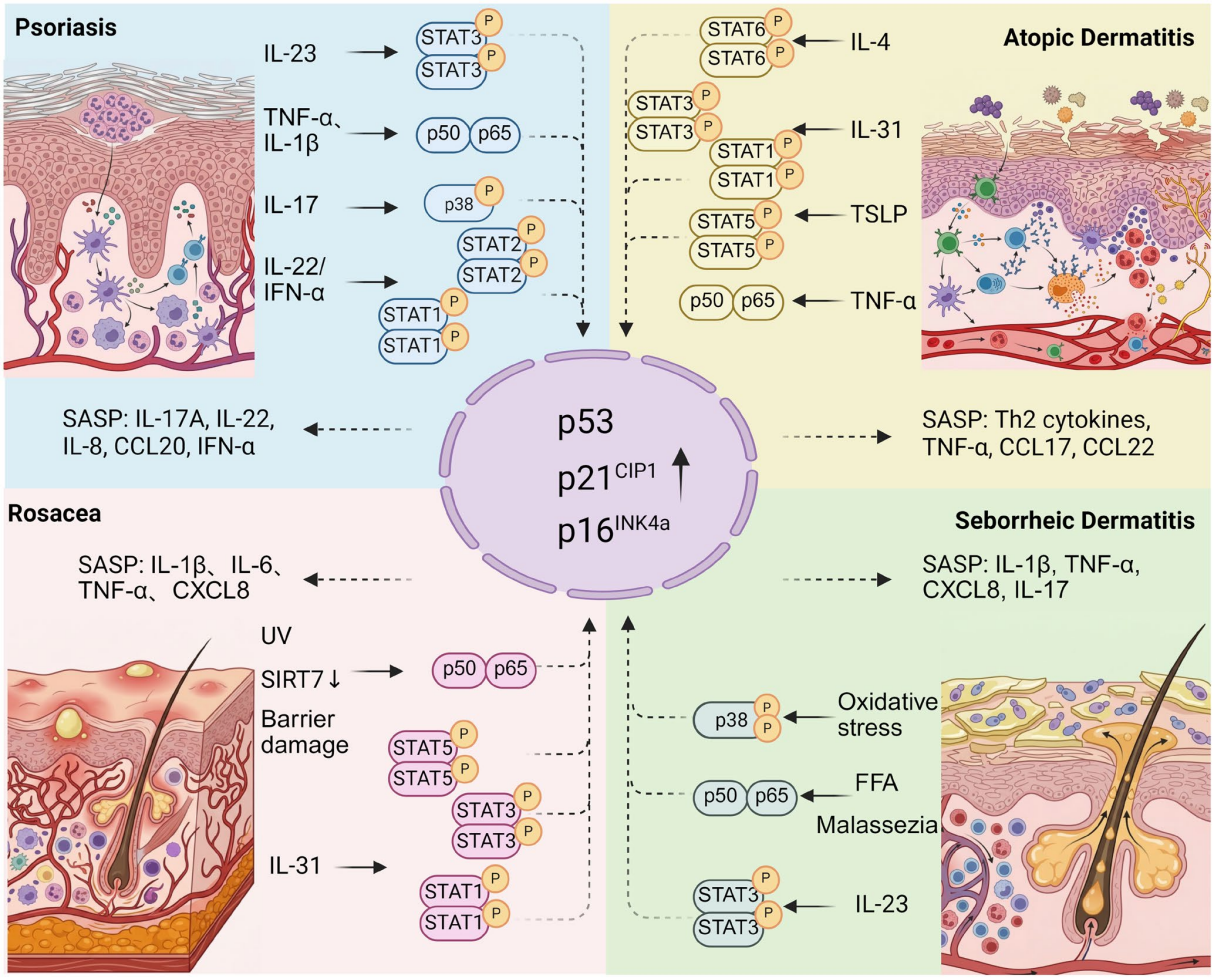
Differences in the involvement of senescent T‐cells across inflammatory skin diseases. Immune‐epithelial cross‐talk mechanisms exhibit distinct spatial and functional segregation. In Psoriasis, a Th17‐skewed SASP from senescent T cells drives aberrant keratinocyte hyperproliferation. In AD, T cell‐derived Th2 SASP directly inhibits epidermal barrier proteins. In Rosacea, potent SASP from senescent T cells triggers keratinocytes to produce LL37, mediating vascular damage. In SD, a bidirectional cascade (keratinocyte‐derived IL‐23 and T cell‐derived IL‐17A/SASP) disrupts epidermal differentiation.

Collectively, while senescent T cells act as central inflammatory drivers across these diverse dermatoses, their specific upstream triggers, resulting SASP profiles, and subsequent immune‐epithelial cross‐talk mechanisms exhibit highly distinct spatial and functional segregation (Figure 5). Understanding these divergent pathogenic cascades provides a critical biological foundation for developing disease‐specific targeted therapies.

## 4 Therapeutic Strategies Targeting T Cell Immunosenescence in Inflammatory Skin Diseases

**FIGURE 6:**
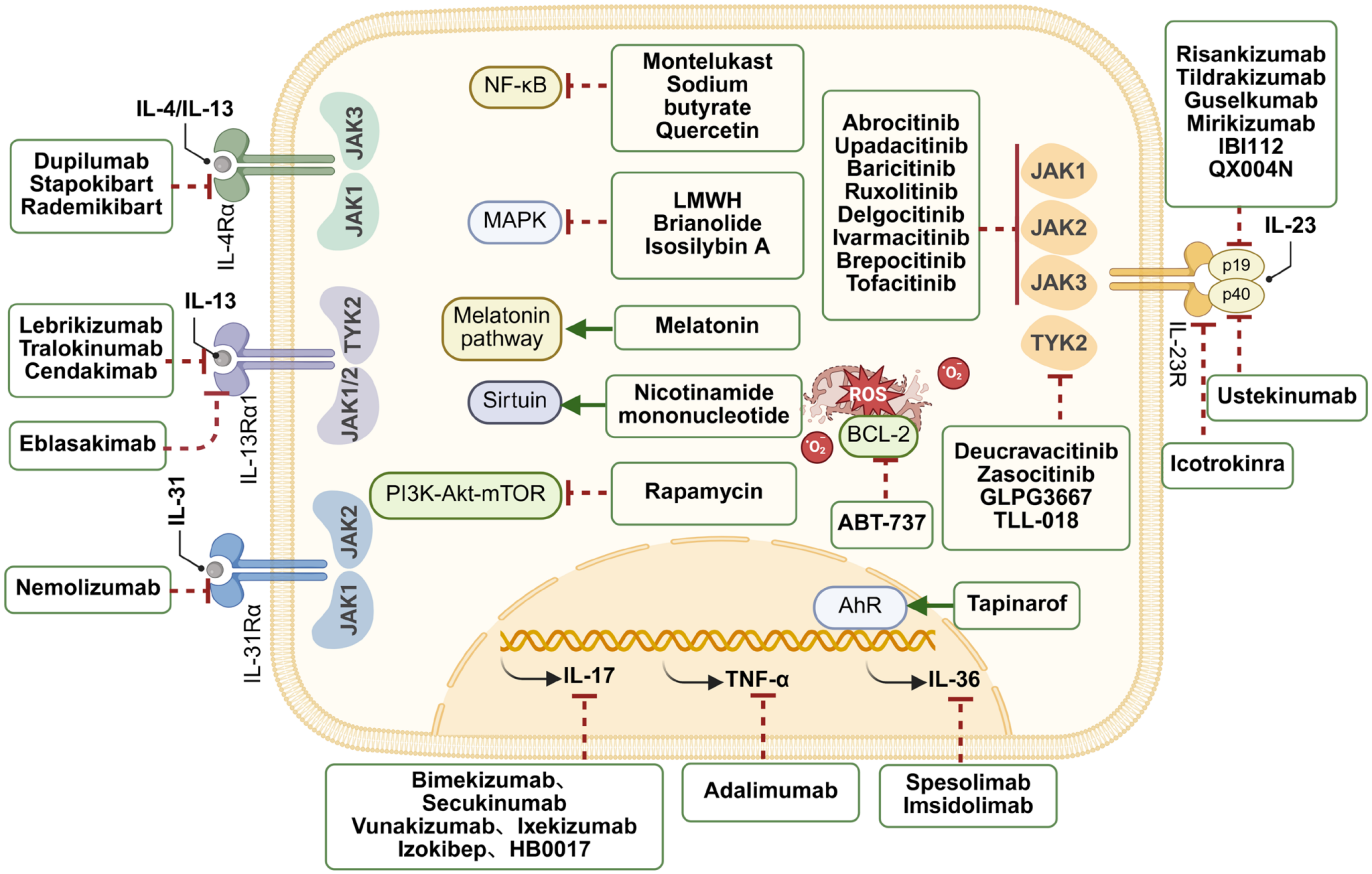
Therapeutic landscape targeting signaling pathways and SASP in immunosenescent T cells. Pharmacological interventions target multiple checkpoints to attenuate T cell senescence and associated inflammation. Extracellularly, biologic agents blockade upstream cytokine receptors, including the interleukin‐4 (IL‐4)/IL‐13 axis (e.g., dupilumab), IL‐31 (nemolizumab), and the IL‐23 pathway (e.g., risankizumab, ustekinumab), preventing downstream signal transduction. Intracellularly, small molecules inhibit specific kinases, including Janus kinase (JAK) family members (JAK1‐3 and tyrosine kinase 2 [TYK2]) and the phosphoinositide 3‐kinase (PI3K)–Akt–mammalian target of rapamycin (mTOR) axis (rapamycin). Metabolic modulators, such as nicotinamide mononucleotide and melatonin, restore sirtuin activity and antioxidant defenses, while B‐cell lymphoma 2 (BCL‐2) inhibitors (ABT‐737) target apoptotic resistance. Downstream, aryl hydrocarbon receptor (AhR) agonists (tapinarof) and the direct neutralization of senescence‐associated secretory phenotype (SASP) mediators—including IL‐17, tumor necrosis factor‐α (TNF‐α), and IL‐36—mitigate the propagated inflammatory response. Red dashed lines indicate inhibition; green arrows indicate activation. (Created with biorender.com).

Inflammatory skin diseases remain challenging to completely cure due to the complexity of their pathogenesis and the chronicity of the disease course. The immune imbalance of the cutaneous microenvironment caused by immunosenescent T cells and their secreted SASP plays a pivotal role in the pathogenesis of inflammatory skin diseases. Currently, an increasing number of biological agents targeting immunosenescent T cells are participating in clinical trials and being applied in clinical practice. Classical targeted therapies include those targeting BCL‐2, NF‐κB, MAPK, and JAK‐STAT signaling pathways as well as SASP components such as IL‐4, IL‐17A, and IL‐23 (Figure 6). Targeted drugs currently approved by the Food and Drug Administration (FDA) or under investigation are systematically summarized in Table 2, with statuses updated as of March 2026.

**TABLE 2:** Summary of interventional targets for immunosenescent T cells in inflammatory skin diseases (Updated to March 2026).

Target	Agent	Major application	Patients included	Drug stage	References and ClinicalTrials.gov identifier
BCL‐2	ABT‐737 gel	Psoriasis	/	Exploratory	Zhu et al. (2024)
PI3K‐Akt‐mTOR	Rapamycin	Psoriasis	/	Exploratory	Bürger et al. (2017)
		AD	/	Exploratory	Jung et al. (2015)
		Rosacea	/	Exploratory	Zeng et al. (2022)
NF‐κB	Montelukast	Psoriasis	/	Exploratory	Zhao et al. (2024)
	sodium butyrate	AD	/	Exploratory	Hu et al. (2024)
	Quercetin	Rosacea	/	Exploratory	Meng et al. (2024)
MAPK	LMWH	Psoriasis	/	Exploratory	Wang, He, et al. (2025)
	brianolide	AD	/	Exploratory	Wang, Wang, et al. (2025)
	Isosilybin A	Rosacea	/	Exploratory	Wu et al. (2024)
Sirtuin	Nicotinamide mononucleotide	Psoriasis	/	Exploratory	Zhang, Cheng, et al. (2024)
Melatonin	Melatonin	Psoriasis	/	Exploratory	Shen et al. (2024)
	Melatonin	Mod‐to‐sev AD (1–18 years)	48	Phase II/III (Completed)	NCT01638234
JAK1	Abrocitinib	Mod‐to‐sev AD	3166	Approved	NCT03422822
	Upadacitinib	Mod‐to‐sev AD	912	Approved	NCT03569293
	Ivarmacitinib	Mod‐to‐sev AD	336	Phase III (Completed)	NCT04875169
JAK1/2	Baricitinib	Mod‐to‐sev AD	1645	Phase III (Completed)	NCT03334435
JAK1/2	Ruxolitinib	Mod‐to‐sev AD (2–12 year)	330	Approved	NCT04921969
		SD	45	Phase II (Completed)	NCT05787860
JAK1/3	Tofacitinib	Erythematotelangiectatic & Papulopustular rosacea	21	Exploratory	Sun et al. (2022)
		Mod‐to‐sev AD	220	Phase IV (Ongoing)	NCT06465732
JAK1/TYK2	Brepocitinib	Mild‐to‐mod AD	292	Phase II (Completed)	NCT03903822
JAK1/2/3, TYK2	Delgocitinib	Chronic hand eczema	513	Approved	NCT05259722
TYK2	Deucravacitinib	Mod‐to‐sev plaque psoriasis	1466	Approved	NCT04036435
	Zasocitinib	Mod‐to‐sev plaque psoriasis	259	Phase II (Completed)	NCT04999839
	GLPG3667	Mod‐to‐sev plaque psoriasis	30	Phase I (Completed)	NCT04594928
TYK2/JAK1	TLL‐018	Mod‐to‐sev plaque psoriasis	73	Phase I (Completed)	NCT05342428
IL‐4Rα	Dupilumab	Mod‐to‐sev AD	71	Approved	NCT03389893
		Mod‐to‐sev AD	188		NCT04033367
		Mod‐to‐sev AD	880		NCT02612454
	Stapokibart	Mod‐to‐sev AD	500	Phase III (Completed)	NCT05265923
	Rademikibart	Mod‐to‐sev AD	226	Phase II (Completed)	NCT04444752
IL‐31	Nemolizumab	Mod‐to‐sev AD	941	Approved	NCT03985943
		Mod‐to‐sev AD	787		NCT03989349
AhR	Tapinarof	Mod‐to‐sev AD (aged > 2 years)	407	Approved	NCT05014568
		Mod‐to‐sev AD (aged > 3 years)	406		NCT05032859
		Mod‐to‐sev AD	728		NCT05142774
		Plaque psoriasis	763	Approved	NCT04053387
IL‐13	Lebrikizumab	Mod‐to‐sev AD	424	Approved	NCT04146363
		Mod‐to‐sev AD	445		NCT04178967
		Mod‐to‐sev AD (6 months–18 years)	310		NCT05735483
		Moderate AD	200		NCT07006792
	Tralokinumab	Mod‐to‐sev AD	802	Approved	NCT03131648
		Mod‐to‐sev AD	794		NCT03160885
		Mod‐to‐sev AD	380		NCT03363854
		Mod‐to‐sev AD (12–18 years)	301		NCT03526861
		Moderate–severe AD (6 months–12 years)	195		NCT06311682
	Cendakimab	moderate–severe AD	221	Phase II (Completed)	NCT04800315
	Eblasakimab	Mod‐to‐sev AD	75	Phase II (Completed)	NCT05694884
IL‐36	Spesolimab	Mod‐to‐sev AD	51	Phase II (Completed)	NCT03822832
		Generalized pustular psoriasis	152	Approved	NCT04015518
	Imsidolimab	Generalized pustular psoriasis	45	Phase III (Completed)	NCT05352893
IL‐23 (p19)	Risankizumab	Mod‐to‐sev plaque psoriasis	2170	Approved	NCT03047395
	Tildrakizumab	Mod‐to‐sev plaque psoriasis	1090	Approved	NCT01729754
	Guselkumab	Mod‐to‐sev plaque psoriasis	992	Approved	NCT02207244
	Mirikizumab	Mod‐to‐sev plaque psoriasis	1484	Phase III (Completed)	NCT03535194
	QX004N	Mod‐to‐sev plaque psoriasis	55	Phase I (Completed)	CTR20212313
	IBI112	Mod‐to‐sev plaque psoriasis	46	Phase I (Completed)	NCT04511624
IL‐23 (p40)	Ustekinumab	Mod‐to‐sev plaque psoriasis	509	Approved	NCT04673786
IL‐23R	Icotrokinra (JNJ‐77242113)	Mod‐to‐sev plaque psoriasis	774	Approved	NCT06143878
IL‐17A/F	Bimekizumab	Mod‐to‐sev plaque psoriasis	1353	Approved	NCT03598790
IL‐17A	Secukinumab	Mod‐to‐sev plaque psoriasis	196	Approved	NCT03020199
	Vunakizumab	Mod‐to‐sev plaque psoriasis	1564	Phase IV (Ongoing)	NCT06779097
	Ixekizumab	Mod‐to‐sev plaque psoriasis	438	Approved	NCT03364309
	Izokibep	Mod‐to‐sev plaque psoriasis	108	Phase II (Completed)	NCT03591887
	HB0017	Mod‐to‐sev plaque psoriasis	40	Phase I (Completed)	NCT04505033
TNF‐α	Adalimumab	Mod‐to‐sev plaque psoriasis	367	Approved	NCT05495568
		Mod‐to‐sev plaque psoriasis	567		NCT04453137

Abbreviations: AD, Atopic dermatitis; AhR, Aryl hydrocarbon receptor; BCL‐2, B‐cell lymphoma 2; IL, Interleukin; JAK, Janus kinase; LMWH, Low molecular weight heparin; MAPK, Mitogen‐activated protein kinase; Mod‐to‐sev, Moderate‐to‐severe; mTOR, Mammalian target of rapamycin; NF‐κB, Nuclear factor‐κB; PI3K, Phosphoinositide 3‐kinase; SD, Seborrheic dermatitis; TNF‐α, Tumor necrosis factor‐α; TYK2, Tyrosine kinase 2.

### 4.1 Targeting BCL‐2

Overexpressed BCL‐2 protein present in psoriatic lesions directly drives inflammation and keratinocyte hyperproliferation of the disease by inhibiting apoptosis and promoting the accumulation of senescent cells (Yildiz et al. 2003). Zhu et al. innovatively applied a topical anti‐aging BCL‐2 inhibitor ABT‐737 gel, which ameliorated imiquimod‐induced psoriasis‐like dermatitis by reducing the proportion of senescent cells in lesions and the expression of the senescence‐associated secretory phenotype SASP, regulating the TCRαβ receptor repertoire, and inhibiting the Tet2‐Th17 pathway (Zhu et al. 2024).

### 4.2 Targeting NF‐κB/MAPK/PI3K‐Akt‐mTOR Pathway

The NF‐κB/MAPK/PI3K‐Akt‐mTOR signaling pathways are the major pathways synergistically amplifying inflammation in the aforementioned inflammatory skin diseases. Accumulating studies indicate that numerous natural compounds can target the above pathways via single or multiple targets to modulate senescence‐driving pathways within T cells. Thatikonda et al. found that Piperlongumine (PPL), the main component of the fruit of *Piper longum* , inhibited PI3K/Akt and NF‐κB pathways by inducing ROS‐mediated mitochondrial apoptosis pathways and inhibiting histone deacetylase (HDAC) activity (Thatikonda et al. 2020). Xu et al. found that Salidroside inhibited MAPK, NF‐κB, and STAT3 pathways in psoriasis‐associated oxidative stress via SIRT1 activation (Xu et al. 2019). Artemisinin suppresses multiple receptor‐coupled signaling pathways, collectively inhibiting multiple key transcription factor pathways dominated by NF‐κB and including PI3K‐Akt‐mTOR, MAPK, and HIF‐1α (Efferth and Oesch 2021). These natural products hold promising application prospects in psoriasis, AD, and rosacea.

### 4.3 Targeting JAK‐STAT Pathway

The JAK family comprises four members: JAK1, JAK2, JAK3, and TYK2. The STAT family consists of seven members: STAT1, STAT2, STAT3, STAT4, STAT5a, STAT5B, and STAT6 (Hu et al. 2021). Biologics such as Abrocitinib, Upadacitinib, Baricitinib, Ruxolitinib, Delgocitinib, Ivarmacitinib, Brepocitinib, and Tofacitinib primarily act on JAK1‐3 subunits and are applied in AD (Reich et al. 2022; Papp, Szepietowski, et al. 2023; Worm et al. 2022; Zhao, Gooderham, et al. 2025; Landis et al. 2022; Sun et al. 2022); while Deucravacitinib, Zasocitinib, GLPG3667, and TLL‐018 highly selectively target the TYK2 subunit (Armstrong et al. 2023, 2024; Mammoliti et al. 2024; Chen et al. 2025), demonstrating greater efficacy in psoriasis. Overall, a large number of Phase III clinical trials have investigated the efficacy of JAK inhibitors (JAKi) in AD and psoriasis, and some JAKi have entered post‐marketing real‐world studies. Safety studies are gradually expanding their indicated age range.

### 4.4 Targeting IL‐4Rα

Dupilumab is a fully human VelocImmune‐derived monoclonal antibody that specifically blocks the common receptor subunit IL‐4Rα of IL‐4 and IL‐13 (Simpson, Schlievert, et al. 2023), thereby inhibiting key signaling pathways of type 2 inflammation. It is currently FDA‐approved for adults and children aged 6 months and older with moderate‐to‐severe disease inadequately controlled with topical therapies. Established Phase IV clinical trials have demonstrated the safety and efficacy of Dupilumab in adults. Compared with placebo, patients treated with Dupilumab showed significant improvement in the sleep quality numerical rating scale (NRS) at week 12 [least squares mean difference (LSMD) −15.5%, *p* < 0.001], as well as statistically significant improvements in peak pruritus NRS (PP NRS), change in SCORing Atopic Dermatitis (SCORAD), and SCORAD sleep visual analogue scale (VAS) (NCT04033367) (Merola, Chiou, et al. 2023). Another clinical trial (NCT03389893) demonstrated that Dupilumab treatment significantly reduced *Staphylococcus aureus* colonization colony counts and cytotoxin levels after only 3 and 7 days, accompanied by reductions in CCL17 and other type 2 biomarkers (Simpson, Schlievert, et al. 2023). Additionally, Phase II/III clinical trials (NCT02407756, NCT02612454, NCT03054428, NCT03346434) have preliminarily demonstrated the efficacy and safety of Dupilumab in children and adolescents aged 6 months to 18 years with moderate‐to‐severe AD (Blauvelt et al. 2022; Paller et al. 2022).

Stapokibart is similarly a humanized monoclonal antibody targeting IL‐4Rα. Compared with Dupilumab, Stapokibart exhibited higher response rates for EASI‐75, AD signs (EASI/IGA/BSA), and symptoms (PP‐NRS) at week 16 of treatment (Zhao, Zhang, et al. 2025; Deleuran et al. 2020). The numerically superior efficacy of Stapokibart may be partially attributed to its unique binding epitope, which binds to IL‐4Rα at a position closer to the ligand‐binding site than Dupilumab, and it has shown comparable or numerically higher potency in blocking IL‐4Rα‐mediated signaling in vitro (Liu et al. 2024).

### 4.5 Targeting IL‐31

Nemolizumab is an antagonist of the IL‐31 receptor subunit α (Silverberg, Wollenberg, et al. 2024). IL‐31, primarily produced by Th2 cells, acts as a key “pruritus factor,” with its receptor predominantly expressed on keratinocytes and sensory nerve fibers. By acting on IL‐31RA to block IL‐31 binding, Nemolizumab rapidly abrogates pruritus generation and progressively improves skin lesions (Silverberg et al. 2020; Kwatra et al. 2023). In two large multinational clinical trials reported in *The Lancet* (NCT03985943, NCT03989349), Nemolizumab demonstrated a rapid onset of action, exhibiting differences from placebo in alleviating pruritus and sleep disturbance as early as week 1, which was faster than Dupilumab and lebrikizumab. However, as the study duration extended to week 16, the response rates for EASI‐75 and IGA with Nemolizumab were inferior to those of the aforementioned biologics (Silverberg, Wollenberg, et al. 2024; Blauvelt et al. 2017; Simpson, Gooderham, et al. 2023).

### 4.6 Targeting AhR

Tapinarof is an aryl hydrocarbon receptor (AhR) agonist that directly binds to and activates AhR, resulting in the downregulation of proinflammatory cytokines (including IL‐17, IL‐22, TNF‐α) and the upregulation of skin barrier proteins. It also activates the nuclear factor erythroid 2‐related factor 2 (Nrf2) pathway, leading to the upregulation of antioxidant enzyme gene expression (Keam 2022), thereby alleviating oxidative stress in the cutaneous microenvironment induced by immunosenescent T cells. The FDA approved it in 2022 for the treatment of plaque psoriasis in adults aged 18 years and older. Currently, an ongoing clinical trial is expanding its indication to pediatric plaque psoriasis (NCT05172726), and two Phase III ADORING trials (NCT05014568, NCT05032859) have demonstrated that 1% Tapinarof cream exhibits highly significant efficacy as well as favorable safety and tolerability in diverse AD patient populations aged 2 years and older (Silverberg, Eichenfield, et al. 2024).

### 4.7 Targeting IL‐13

Lebrikizumab is a novel high‐affinity monoclonal antibody that selectively binds IL‐13, preventing the formation of the IL‐13Rα1/IL‐4Rα heterodimer signaling complex, thereby blocking the biological activity of IL‐13 (Silverberg et al. 2023). In two Phase III monotherapy studies, ADvocate 1 & 2 (NCT04146363, NCT04178967), induction with Lebrikizumab every 2 weeks followed by dosing every 4 weeks was sufficient to maintain remission in the majority of patients with moderate‐to‐severe AD (Silverberg et al. 2023; Blauvelt, Thyssen, et al. 2023). The long‐term effects may be attributed to the long half‐life of Lebrikizumab (25 days) (Zhu et al. 2017) as well as its high binding affinity for IL‐13, low dissociation rate, and higher potency than tralokinumab (Blauvelt, Thyssen, et al. 2023; Wollenberg et al. 2021). Currently, Phase III (NCT05735483, 6 months to 18 years) and Phase IV (NCT07006792, adolescents and adults) clinical trials are recruiting to expand the patient population for Lebrikizumab.

Tralokinumab is a fully human IgG4 monoclonal antibody that binds IL‐13 with high affinity (Paller et al. 2023). The ECZTRA series of clinical trials first confirmed the efficacy and safety of Tralokinumab as monotherapy (without TCS) in the treatment of moderate‐to‐severe AD in adults (NCT03131648, NCT03160885) (Wollenberg et al. 2021). Building on this, long‐term assessments of efficacy and safety were conducted for combination therapy with topical TCS in adults with moderate‐to‐severe AD (NCT03363854) (Silverberg et al. 2021) and in adolescents aged 12–18 years with moderate‐to‐severe AD (NCT03526861) (Paller et al. 2023), supporting its therapeutic value. An ongoing recruiting Phase III clinical trial (NCT06311682) extends the application of Tralokinumab to infants and children aged 6 months to 12 years, with its safety and efficacy pending validation by the results.

Additionally, there are two other biologics targeting IL‐13: Cendakimab directly binds IL‐13, blocking the interaction of IL‐13 with its receptors IL‐13Rα1 and IL‐13Rα2 (Blauvelt et al. 2024); while Eblasakimab binds IL‐13Rα1 with high affinity, preventing the formation of the IL‐13Rα1/IL‐4Rα heterodimeric receptor signaling complex (Veverka et al. 2024). Significant reductions in Eczema Area and Severity Index scores have been observed for both in current Phase II clinical trials (NCT04800315, NCT05694884) (Blauvelt et al. 2024; Veverka et al. 2024).

### 4.8 Targeting IL‐23

Risankizumab, Tildrakizumab, Guselkumab, and Mirikizumab specifically inhibit IL‐23 by binding to the p19 subunit of IL‐23 (Papp, Blauvelt, et al. 2023; Gebauer et al. 2024; Schäkel et al. 2023; Papp, Warren, et al. 2023); Icotrokinra selectively blocks the interleukin‐23 receptor (Bissonnette et al. 2024); while Ustekinumab binds to the p40 protein subunit shared by the IL‐12/23 heterodimer (Szepietowski et al. 2025). Recent research by Wambier et al. found that the mean epigenetic age of patients with psoriasis is 5 years older than their corresponding chronological age. A Phase IV clinical trial by this team (NCT05110313) investigates whether Tildrakizumab can reverse peripheral blood leukocyte DNA methylation associated with chronic psoriasis, namely epigenetic aging (data not yet published). The results of this study hold promise for treating psoriasis from the perspective of targeting immunosenescence.

### 4.9 Targeting IL‐17A

With the exception of Bimekizumab, which simultaneously targets IL‐17A/F (Merola, Landewé, et al. 2023), other biologics such as Secukinumab, Vunakizumab, and Ixekizumab highly selectively target IL‐17A (Iversen et al. 2023; Yan et al. 2025; Gao et al. 2025). A Phase IIa clinical trial (NCT03553823) demonstrated that Secukinumab exerted superior clinical and molecular effects compared to Guselkumab in Ustekinumab‐refractory psoriatic plaques. This may be attributed to the presence of an IL‐23‐independent inflammatory drive in non‐responsive lesions, rendering the direct targeting of the downstream terminal effector IL‐17A more advantageous than upstream IL‐23 blockade (Krueger et al. 2023).

## 5 Challenges and Future Prospects

In conclusion, the signaling network within immunosenescent T cells encompassing NF‐κB, JAK–STAT, and mTOR functions as the core engine driving disease recurrence and chronicity by establishing a senescence‐inflammation positive feedback loop. This network not only arrests the cell cycle via the p53‐p21 pathway but also precisely programs the SASP centered on IL‐17, IL‐4, and TNF‐α, which continuously exacerbates the local microenvironment and reciprocally reactivates pathogenic pathways to form a self‐sustaining vicious cycle (Wang, Han, Elisseeff, and Demaria 2024; Fu et al. 2025). Consequently, disrupting this cycle constitutes the theoretical foundation for therapeutic intervention.

Given the high interconnectivity of this network, current clinical development focuses on blocking critical signaling hubs such as the JAK–STAT pathway or intercepting downstream SASP effectors (Armstrong and Read 2020; Alvarenga et al. 2024). However, the varying contributions of specific signaling nodes across different disease contexts dictate the specificity of targeted drug selection and the heterogeneity of clinical outcomes (Guttman‐Yassky and Krueger 2017; Hren et al. 2024; Gottlieb 2005). Crucially, while existing therapies significantly alleviate symptoms, they are largely limited to downstream interception and have yet to fundamentally reverse the upstream immunosenescent phenotype.

Regarding JAK inhibitors (JAKi), in AD, IL‐4 binds to the type I IL‐4R, stimulating the phosphorylation of JAK1 and JAK3, which subsequently activates and phosphorylates IL‐4Rα and STAT6 (Miyazaki et al. 1994); phosphorylated STAT6 then dimerizes and acts as a transcription factor by binding to specific DNA sequences of IL‐4‐responsive genes (Huang et al. 2022), driving inflammation and barrier disruption. In psoriasis, IL‐23 binds to the receptor complex composed of IL‐23Rα and IL‐12Rβ1, primarily stimulating the phosphorylation of JAK2 and TYK2, which subsequently preferentially activates and phosphorylates STAT3, thereby driving the Th17 pathway (Lee et al. 2004; Kamata and Tada 2022; Mylonas and Conrad 2018), representing the core pathogenic link in psoriasis. Consequently, JAK1‐targeting agents such as Abrocitinib and Upadacitinib are primarily applied in the treatment of AD, whereas TYK2‐targeting agents such as Deucravacitinib and Zasocitinib are primarily applied in psoriasis. Beyond these core pathways, in psoriasis, IFN‐α and IFN‐β stimulate the phosphorylation of JAK1 and TYK2, while IFN‐γ stimulates the phosphorylation of JAK1 and JAK2 (Gómez‐García et al. 2022; Sugumaran et al. 2024). Upadacitinib, as a biologic targeting JAK1, is widely applied in AD; furthermore, numerous clinical trials have demonstrated its efficacy and safety in psoriatic arthritis (McInnes et al. 2021; Burmester et al. 2023; Mease et al. 2021), yet there are currently no studies on its application in psoriasis. Tofacitinib targets JAK1/3, with Phase III clinical trials (NCT01186744, NCT01815424, NCT01163253) demonstrating its efficacy in moderate‐to‐severe plaque psoriasis (Bissonnette et al. 2015; Zhang et al. 2017; Valenzuela et al. 2018); however, recent clinical trials have focused on its application in psoriatic arthritis (Gladman et al. 2024, 2025), and expanding its clinical indication to plaque psoriasis still requires further data support.

Dupilumab targeting IL‐4Rα and JAK inhibitors (JAKi) targeting the JAK–STAT pathway are the most widely applied agents in AD. Multiple head‐to‐head studies (NCT04345367, NCT03738397, NCT05601882) comparing their efficacy in AD indicate that abrocitinib/upadacitinib are more effective than dupilumab in improving pruritus and symptoms in moderate‐to‐severe AD (Reich et al. 2022; Silverberg, Bunick, et al. 2024). Patients who previously had an inadequate response to dupilumab or discontinued due to significant side effects achieved improvements in skin clearance and pruritus reduction after using upadacitinib (Blauvelt, Ladizinski, et al. 2023). Currently, the most likely explanation is that JAKi affect the signal transduction of multiple immune and epidermal cell‐derived cytokines (including TSLP, IL‐4, IL‐13, IL‐22, IL‐31, etc.) (Huang et al. 2022), whereas the direct action of dupilumab is mainly limited to IL‐4 and IL‐13 (Simpson, Schlievert, et al. 2023). In contrast, in psoriasis (NCT06143878, NCT06220604), icotrokinra demonstrated superior clinical response rates compared to the JAK inhibitor deucravacitinib (Gold et al. 2025), which may be related to icotrokinra specifically targeting the most core IL‐23 in the upstream pathogenesis (Bissonnette et al. 2024), while the action of deucravacitinib is more dispersed.

Current research targeting SASP factors and signaling pathways is predominantly focused on psoriasis and AD, whereas investigations in rosacea and SD are primarily limited to case reports. A clinical study by Sun Y et al. involving 21 patients with rosacea demonstrated that 71.4% of patients achieved a significant reduction in facial erythema following oral tofacitinib treatment (Sun et al. 2022). Xu B and Zhang T respectively reported that 4 patients with steroid‐induced rosacea treated with Abrocitinib and 2 patients with refractory rosacea treated with Upadacitinib achieved symptomatic improvement (Xu et al. 2023; Zhang, Liu, et al. 2024). Sahoo CK et al. recently reported a case series of 12 adult patients with severe to very severe SD treated with oral tofacitinib, where a three‐month follow‐up indicated significant improvement in pruritus symptoms, and all patients are currently continuing follow‐up (Sahoo et al. 2026). Additional clinical observation reports of patients with comorbid psoriasis treated with secukinumab or guselkumab showed simultaneous improvement of SD (Mital et al. 2023); upadacitinib, abrocitinib, and baricitinib have also been proven effective in patients with overlapping AD/SD features (He et al. 2024), suggesting that biologics currently widely applied in psoriasis and AD hold promise for expanding their indications to inflammatory skin diseases such as rosacea and SD due to the commonality of pathways and targets.

Currently, treatments for inflammatory skin diseases are concentrated on SASP factors, whereas therapies directly targeting immunosenescent T cells have not yet entered the stage of clinical trials. Ruan P et al. found that caffeine intake reduced the frequency of senescent CD4 + CD57+ T cells in vitro, downregulated JAK/STAT and MAPK pathway activity, decreased SASP levels, and alleviated inflammation and immunosenescence in mice with imiquimod (IMQ)‐induced psoriasis‐like dermatitis (Ruan et al. 2024); Chen Y et al. subjected mice to time‐restricted feeding, which reduced the number of CD4+ senescent T cells and the expression of p21/p16 in the dermis and spleen, simultaneously decreased the quantity of Th2 and Th17 cells in the spleen, and alleviated IMQ‐induced scaling in psoriasis‐like mice (Chen et al. 2023). These latest studies explore the therapeutic potential of directly targeting immunosenescent T cells through animal experiments.

This review systematically elucidates that immunosenescent T cells and their internal signaling networks such as NF‐κB, JAK–STAT, and mTOR are the core mechanisms driving the chronicity and recurrence of various inflammatory skin diseases such as psoriasis and AD. Although current biologic therapies targeting downstream cytokines are effective, it remains difficult to eradicate the pathological cycle maintained by the senescent cells themselves. Therefore, future direct targeted elimination of senescent T cells or precise intervention in their upstream regulatory hubs will be key frontier directions for achieving deep disease remission and overcoming therapeutic resistance.

## Author Contributions

Conghui Liu wrote and revised the manuscript. Ming Yang, Fugang Xiao, and Jinrong Zeng revised the manuscript and provided crucial advice. All authors read and approved the final paper.

## Funding

This work was supported by the Hunan Provincial Health Commission Health Research Key Project (Grant No. 20256627), Science and Technology Innovation Program of Hunan Province (Grant No. 2024RC3064), Wisdom Accumulation and Talent Cultivation Project of the Third Xiangya Hospital of Central South University (Grant No. YX202214), Young Backbone Talent Project of Hunan Provincial Health Commission (Grant No. RC020031).

## Conflicts of Interest

The authors declare no conflicts of interest.

## Data Availability

Data sharing not applicable to this article as no datasets were generated or analysed during the current study.
